# Intensified treatment of hyperphosphatemia associated with reduction in parathyroid hormone in patients on maintenance hemodialysis

**DOI:** 10.1080/0886022X.2017.1419966

**Published:** 2018-01-02

**Authors:** Lan Chen, Jin-Xuan He, Ying-Ying Chen, Yi-Sheng Ling, Chun-Hua Lin, Tian-Jun Guan

**Affiliations:** Department of Nephrology, Zhongshan Hospital, Xiamen University, Xiamen, Fujian, China

**Keywords:** Serum phosphorus, hyperphosphatemia, chronic kidney disease, hemodialysis, parathyroid hormone

## Abstract

**Background:** This study investigated the therapeutic effect of intensive phosphorus-lowering therapy on intact-parathyroid hormone (iPTH) levels in hemodialysis patients.

**Methods:** Ninety-five hemodialysis patients with serum phosphorus ≥1.78 mmol/L and iPTH ≥300 pg/dL were apportioned to either the treatment or control group (*n* = 43 and 52, respectively) based on patient commitment to treatment. The treatment group was given phosphorus-lowering therapies with phosphate binders (lanthanum, sevelamer or/and calcium reagent) combined with dietary phosphate restriction and intensified hemodialysis. The control individuals were given low doses of calcium agents, if serum calcium was <2.54 mmol/L. Percent changes in serum phosphorus and iPTH levels were compared between the two groups. In addition, based on the time required to achieve >20% decrease in serum phosphorus, the patients in the treatment group were further stratified as rapid responders (≤2 months; 27 patients) or slow responders (>2 months; 16 patients) and percent changes in iPTH were compared.

**Results:** Serum phosphorus and iPTH levels decreased from baseline in the treatment group (−24.08 ± 1.93% and −9.92 ± 3.70%, respectively) but increased in the control group (22.00 ± 3.63% and 104.21 ± 23.89%; both *p* < .001). In the rapid responders subgroup, the iPTH decreased (−16.93 ± 3.49%), but in the slow responders subgroup the iPTH increased slightly (0.68 ± 7.37%, *p* < .05).

**Conclusions:** For these patients on maintenance hemodialysis, intensive treatment of hyperphosphatemia was associated with a decrease in iPTH levels, especially for those who had achieved substantial reduction in serum phosphorus within 2 months.

## Introduction

Chronic kidney disease has become a major health concern with a high prevalence worldwide. It is estimated that in China, nearly one in 10 people has some degree of kidney dysfunction, affecting almost 150 million individuals [1]. A common consequence of chronic kidney disease is secondary hyperparathyroidism, or excessive serum parathyroid hormone (PTH), which is largely attributed to the retention of phosphate [2,3].

The etiology of secondary hyperparathyroidism is complex and multifactorial, while hyperphosphatemia and abnormal bone remodeling are associated with poor clinical outcomes [4–9]. Streja et al. [10] reported that in patients on maintenance hemodialysis, hyperphosphatemia is associated with both high serum PTH levels and excessive dietary protein intake. Martin et al. [11] found that dietary phosphate or intravenous infusion of sodium phosphate could increase serum PTH within 10 min, without change in plasma calcium. Other studies have also found that plasma PTH levels change in response to dietary phosphate intake [13]. It seems that dietary phosphate can regulate parathyroid gland function, independent of changes in serum calcium or intake of vitamin D sterol (1,25**-**dihydroxyvitamin D_3_, or 1,25[OH]2D3) [12]. Furthermore, elevated serum phosphorus levels may increase the risk of obstructive atherosclerotic coronary artery disease in non-chronic kidney disease patients [14–16].

In the present study, we explored whether a reduction in serum phosphorus could reduce iPTH levels, in the absence of vitamin D sterol treatment. We screened patients with hyperphosphatemia and secondary hyperparathyroidism, who had been diagnosed in accordance with the guidelines of the Kidney Disease Improving Global Outcomes (KDIGO). The inclusion criteria were serum phosphorus ≥1.78 mmol/L [17] and iPTH ≥300 pg/dL [18,19]. We then investigated an association between the level of intact-PTH (iPTH) and serum phosphorus in patients being given maintenance hemodialysis. On the other hand, in China the most effective dietary phosphorus binders are expensive but are not covered by Chinese medical insurance. So the data presented in this study maybe therefore urgently needed as evidence to persuade the relevant authorities to allow medical insurance to cover dietary phosphorus binders.

## Methods

The Human Research Ethics Board at Xiamen Zhongshan Hospital approved this study. All patients provided written informed consent.

### Study population

**Figure 1.**
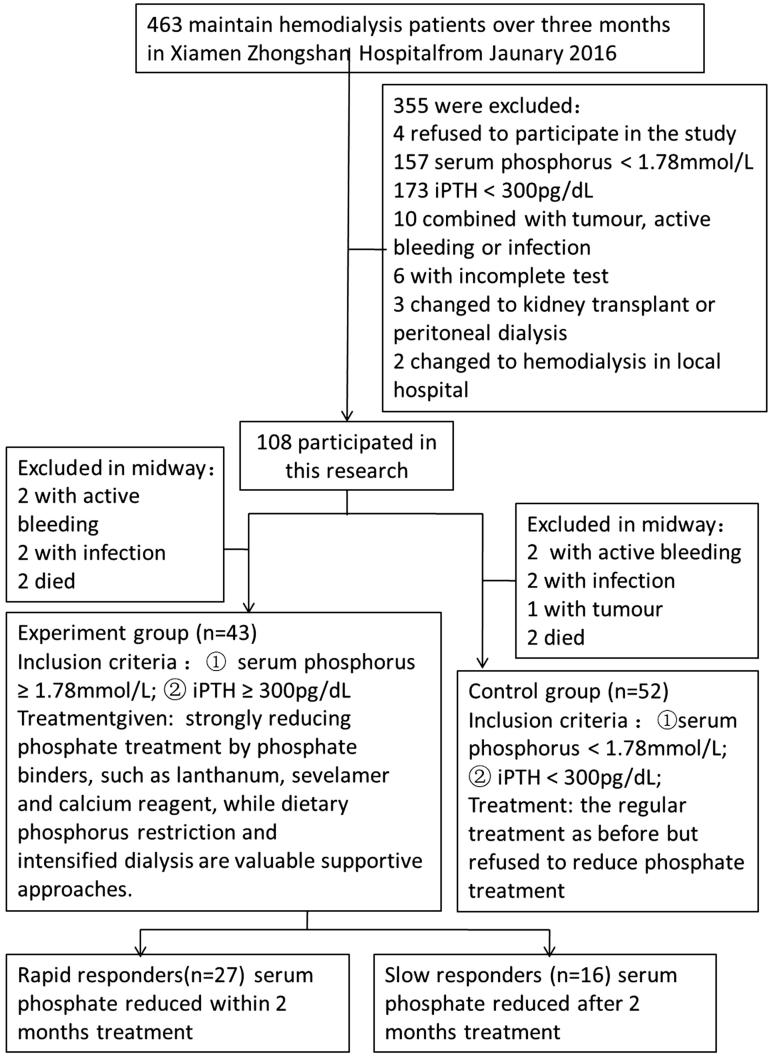
Flow chart of the study.

From 1 February 2016 to 31 July 2016, 95 patients with stage-5 chronic kidney disease and on maintenance hemodialysis were included in this prospective study at Xiamen Zhongshan Hospital, China (Figure 1). All these patients met the following criteria: uremic, on maintenance hemodialysis ≥3 months, serum phosphorus ≥1.78 mmol/L, iPTH ≥300 pg/dL and willing to participate in the study.

Patients with any of the following were excluded from this study: pacemaker or implanted defibrillator, amputation or metallic prosthesis; active bleeding, tumor or infection; incomplete clinical or biochemical data; scheduled for kidney transplantation, transfer to peritoneal dialysis or change of hemodialysis site to a local hospital within 6 months prior to the study.

### Grouping and treatment

Phosphorus-lowering therapy was assigned to patients based on their willingness to adhere to this treatment. Thus, the patients were apportioned to either a treatment group (*n* = 43) or control group (*n* = 52). The treatment group was given phosphorus-lowering therapies with phosphate binders (lanthanum, sevelamer or/and calcium reagent) in combination with supportive approaches that included dietary phosphate restriction and intensified hemodialysis. The total observation period was 6 months. Based on the time required to achieve >20% decrease in serum phosphorus, according to the response time, the patients in the treatment group were further stratified into rapid responders (≤2 months; 27 patients) and slow responders (>2 months; 16 patients) treatment subgroups.

The patients in the control group were given low doses of calcium agents if their serum calcium level was <2.54 mmol/L. None of the patients in the study received any active vitamin D sterol therapy such as calcitriol, calcimimetics or paricalcitol. All the patients were treated routinely in accordance with the KDIGO/KDOQI guidelines in the event of a complication, including anemia, electrolyte abnormalities, CKD-MBD, infection, vascular access complication and so on [17,19].

### Collection of clinical and biochemical data

Data were obtained from a Jinshida computer database system at Xiamen Zhongshan Hospital, which recoded the clinical data of all patients. The following data were collected: age, gender and diagnosis of primary diseases. Biochemical parameters were measured and collected before the midweek dialysis session, and included the following: blood urea nitrogen, serum creatinine, hemoglobin, calcium, phosphorus, albumin and iPTH.

### Statistical analysis

Data were analyzed using SPSS statistical software 22.0 (SPSS, Chicago, IL). Quantitative data are expressed as mean ± standard error of the mean, and the categorical variables are shown as absolute number and percentage. Continuous variables were compared using a paired *t*-test and independent-samples *t*-tests, and categorical variables using the chi-squared test or Fisher’s exact test where appropriate, to evaluate inter-group and intra-group differences. *p* < .05 was considered statistically significant.

## Results

### Characteristics of the patients

A total of 95 uremic patients (66 men and 29 women) on regular hemodialysis were included in this study (Table 1). The average age of patients was 52.19 ± 1.42 years (range: 20–84 years).

**Table 1.** Characteristics of the study population.

	Total	Treatment	Control	*p* value
Subjects, *n*	95	43	52	–
Age, y	52.2 ± 1.4	50.1 ± 2.1	53.9 ± 1.9	.62
Male, *n* (%)	66 (69.5)	28 (65.1)	38 (73.1)	.95
Etiology, *n* (%)				
Glomerulopathy	35 (36.8)	16 (37.2)	19 (36.5)	.67
Diabetes mellitus	26 (27.4)	12 (27.9)	14 (26.9)	–
Hypertension	14 (14.7)	6 (14.0)	8 (15.4)	–
Lupus nephritis	4 (4.2)	2 (4.6)	2 (3.8)	–
ADPKD	4 (4.2)	2 (4.6)	2 (3.8)	–
Gouty nephropathy	4 (4.2)	2 (4.6)	2 (3.8)	–
Vasculitis nephritis	3 (3.2)	2 (4.6)	1 (1.9)	–
Obstructive nephropathy	1 (1.1)	0 (0)	1 (1.9)	–
Chronic allograft nephropathy	2 (2.1)	1 (2.3)	1 (1.9)	–
Tumor associated nephropathy	1 (1.1)	0 (0)	1 (1.9)	–
Unknown causes	1 (2.1)	0 (0)	1 (1.9)	–
Serum calcium, mmol/L				
Pre-treatment	–	2.27 ± 0.03	2.30 ± 0.03	.44
Post-treatment	–	2.28 ± 0.03	2.24 ± 0.03	.31
*p* value	–	.75	.15	–
Serum phosphorus, mmol/L				
Pre-treatment	–	2.66 ± 0.07	2.29 ± 0.06	<.001
Post-treatment	–	2.02 ± 0.07	2.74 ± 0.08	<.001
Rate of change, %	–	−24.08 ± 1.93	22.00 ± 3.63	<.001
*p* value	–	<.001	<.001	–
iPTH, pg/dL				
Pre-treatment	–	741.81 ± 78.35	557.28 ± 66.97	.07
Post-treatment	–	638.93 ± 63.20	871.05 ± 84.37	.03
Rate of change, %	–	−9.92 ± 3.70	104.21 ± 23.89	<.001
*p* value	–	.30	.004	–
Albumin, g/L				
Pre-treatment	–	42.45 ± 0.57	43.11 ± 0.53	.44
Post-treatment	–	43.10 ± 0.54	43.28 ± 0.52	.81
*p* value	–	.41	.82	–
BUN, mmol/L				
Pre-treatment	–	28.85 ± 0.91	27.87 ± 0.96	.46
Post-treatment	–	26.30 ± 1.13	28.61 ± 0.93	.12
*p* value	–	.08	.58	–
SCr, μmol/L				
Pre-treatment	–	1139.39 ± 33.17	1091.68 ± 37.79	.35
Post-treatment	–	1105.81 ± 37.20	1077.87 ± 30.51	.56
*p* value	–	.50	.78	–
Hemoglobin, g/L				
Pre-treatment	–	114.88 ± 3.49	113.40 ± 3.09	.75
Post-treatment	–	111.33 ± 3.16	117.23 ± 2.87	.17
*p* value	–	.45	.37	–

ADPKD: autosomal dominant polycystic kidney disease; BUN: blood urea nitrogen.

The main causes of end-stage renal disease in these patients were the following: primary glomerulopathy (35 patients; 36.8%), diabetic nephropathy (26, 27.4%) and hypertension (14, 14.7%). In addition, four (4.21%) patients each had lupus nephritis, autosomal dominant polycystic kidney disease or gouty nephropathy; three (3.16%) patients had vasculitis-related renal damage; two patients (2.11%) had chronic allograft nephropathy and one (1.05%) patient each had obstructive nephropathy, tumor-associated nephropathy or were idiopathic. There were no significant differences between the treatment and control groups with regard to age, gender, primary diseases or plasma levels of sodium and potassium.

### Serum phosphorus of the treatment and control groups

**Figure 2.**
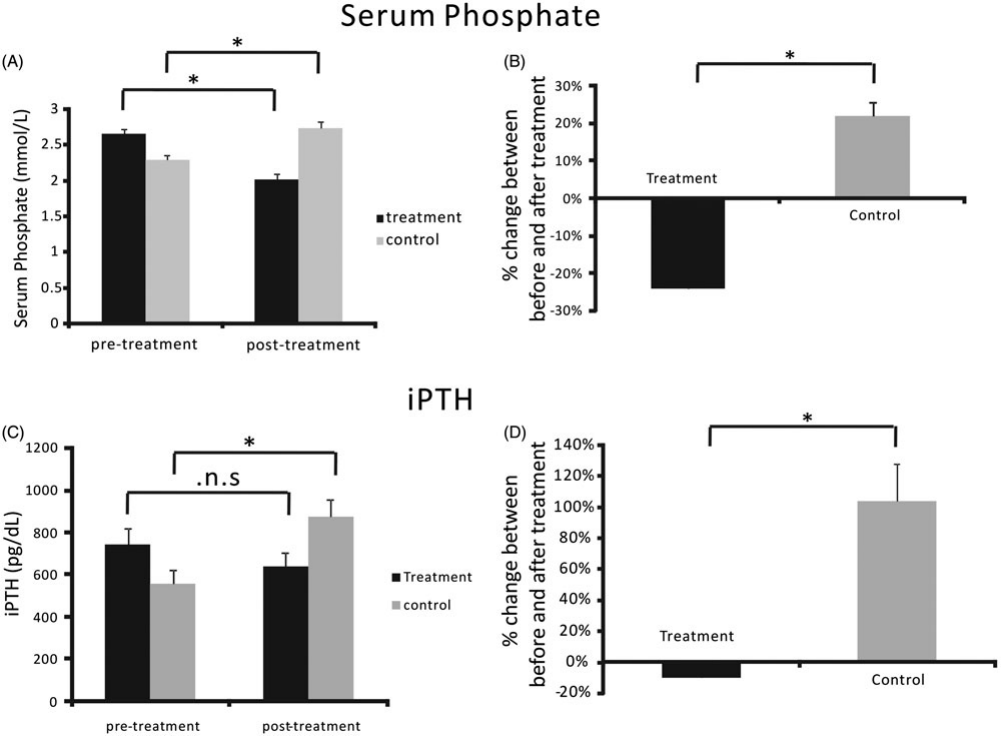
Serum phosphorus and iPTH before and after phosphorus-lowering therapy. (A) Serum phosphorus was significantly lower in the treatment group compared with the control group. (B) Percent change in serum phosphorus after treatment relative to the baseline. (C) iPTH was significantly lower in the treatment group compared with the control group. (D) Percent change in iPTH level after treatment relative to the baseline. *p < .05.

In the 43 patients of the treatment group, the mean serum phosphorus at baseline (pre-treatment; 2.66 ± 0.0665 mmol/L) was significantly lower after the intensive phosphorus-lowering treatment (2.02 ± 0.0731 mmol/L; *p* < .001; Table 1, Figure 2(A)). In the 52 patients of the control group, the mean serum phosphorus at baseline (2.29 ± 0.0644 mmol/L) was significantly higher at the post-treatment timepoint (2.74 ± 0.0821 mmol/L; *p* < .001).

The percent change in serum phosphorus from baseline to post-treatment was significantly different between the treatment (−24.08 ± 1.93%) and control groups (22.00 ± 3.63%; *p* < .001; Table 1, Figure 2(B)). In both groups, the following parameters after treatment were statistically similar to the respective baseline measurements: serum calcium, albumin, hemoglobin, blood urea nitrogen and serum creatinine.

### iPTH levels of the treatment and control groups

In the 43 patients of the treatment group, the pre-treatment iPTH (741.81 ± 78.35 pg/dL) was lower after treatment (638.93 ± 63.20 pg/dL; Table 1, Figure 2(C)). In the 52 patients of the control group, the pre-treatment iPTH (557.28 ± 66.97) was higher after the treatment period (871.05 ± 84.37 pg/dL; *p* < .01).

The percent changes in iPTH levels before and after treatment were significantly different between the treatment group (−9.92 ± 3.70%) and the control group (104.21 ± 23.89%; *p* < .001; Figure 2(D)).

### iPTH levels of the rapid responders and the slow responders treatment groups

**Figure 3.**
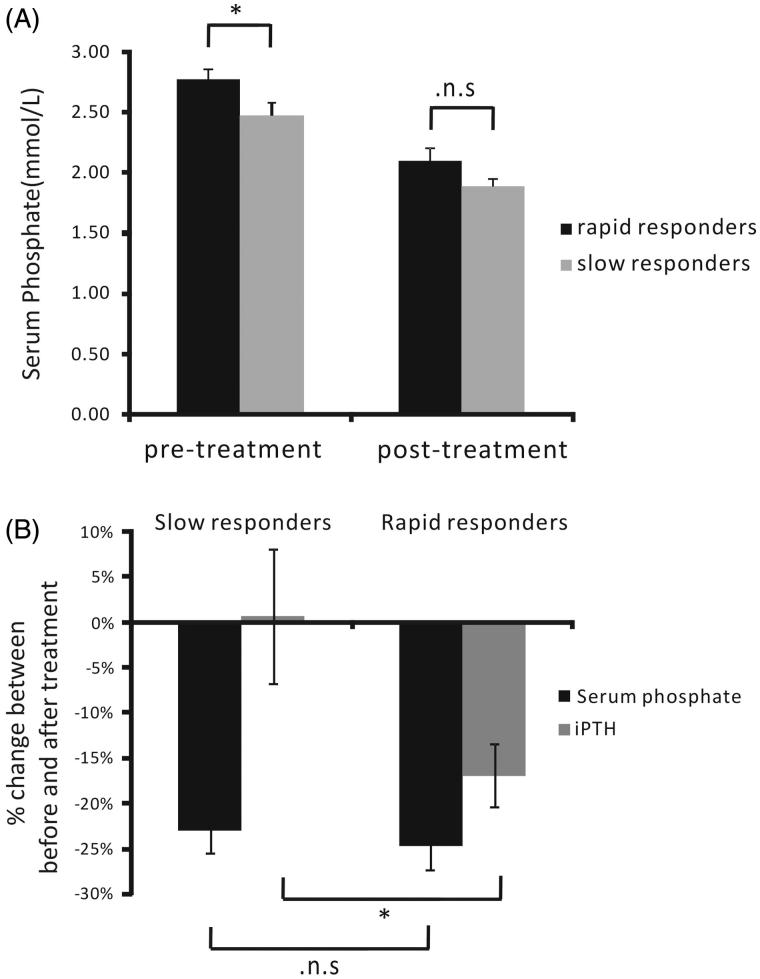
Percent changes in serum phosphorus and iPTH between the rapid responders and the slow responders treatment subgroups. (A) The basal serum phosphorus concentrations before treatment of the rapid responders was significant higher than the slow responders subgroups (*p* = .0275, A). (B) The percent of change in serum phosphorus was −23.07 ± 2.45% in the rapid responders treatment subgroup and −24.68 ± 2.73% in the slow responders treatment subgroup (*p* > .05). The percent of change in iPTH in the rapid responders treatment subgroup was −16.93 ± 3.49%, in contrast with 0.68 ± 7.37% in the slow responders treatment subgroup. *p < .05.

Based on the time required to achieve >20% decrease in serum phosphorus, the patients in the treatment group were further stratified as rapid responders (≤2 months; 27 patients) and slow responders (>2 months; 16 patients). The basal serum phosphorus concentrations and the percent changes in iPTH were compared. The basal serum phosphorus concentrations before treatment between the rapid responders (2.77 ± 0.0816 mmol/L) and the slow responders (2.48 ± 0.101 mmol/L) subgroups were statistically significant (*p* = .0275, Figure 3(A)). The percent changes in serum phosphorus between the rapid responders (−23.07 ± 2.45%) and the slow responders (−24.68 ± 2.73%) treatment subgroups were not statistically significant (*p* = .660, Figure 3(B)). However, there was a significant difference in the percent changes in iPTH levels between the rapid responders (−16.93 ± 3.49%) and the slow responders (0.68 ± 7.37%, *p* = .042) treatment subgroups (Figure 3(B)).

## Discussion

Inappropriate phosphorus retention has a central role in the pathophysiology of mineral and bone disorders in patients with chronic kidney disease, highlighting the clinical significance of serum phosphorus control in such patients. Unfortunately in China, the importance of management and treatment of hyperphosphatemia has not been recognized by many patients for several reasons. Firstly, clinical manifestations of mineral and bone disorders such as bone pain, itchy skin and angiosteosis may not occur for 2–3 years, and patients are without symptoms at the early stage. Secondly, many phosphorus binders are expensive and most of them are not covered under Chinese medical insurance. This results in less selection for many patients, due to a cost that is beyond their ability to pay. Calcium-containing phosphate binders are more economical, but long-term increased dosage of calcium-containing phosphate binders is not recommended, especially in patients with high serum calcium-phosphorus product, where these agents should be used with caution. Therefore, in this study low-dosage of calcium agents were prescribed for those with low serum calcium levels. Thirdly, some patients do not pay attention to dietary phosphate restrictions. In the present study, the patients were divided into two groups, treatment or control, based on their willingness to commit to the treatment regime. The mean baseline serum phosphorus of the treatment group was significantly higher than that of the control group, which may explain the good adherence of these patients to phosphorus-lowering therapy.

In the present study, the therapeutic interventions consisted of dietary phosphate restriction, the use of phosphorus binders and intensified hemodialysis. Based on the time required to achieve a >20% decrease in serum phosphorus, we subdivided the patients in the treatment group into the rapid responders and the slow responders treatment subgroups. Our findings indicate that reduction in high serum phosphorus would help to decrease iPTH levels in patients on maintenance hemodialysis, especially for those rapid responders who achieved a substantial reduction in serum phosphorus within 2 months.

The mechanism by which serum phosphorus regulates secondary hyperparathyroidism remains largely unknown. One hypothesis is that serum phosphate may regulate the expression of the calcium-sensing receptor by the parathyroid glands, as evidenced by the reduced presence of the calcium-sensing receptor in the parathyroid glands of patients with chronic renal failure [20–23]. Our data suggest an interplay between serum phosphorus and IPTH levels. A number of *in vivo* studies have also implied acute regulation of PTH *via* dietary intake of phosphate [11–13]. Our study showed that in patients under treatment who were able to reduce serum phosphorus in a relatively short time, the management of hyperphosphatemia and lowering iPTH levels was more favorable. Our study showed that the basal serum phosphorus in the slow responders was already lower than in the rapid responders group. May be it could explained that the slow responders respond more slowly and the patients would pay less attention to the treatment. Slow responders did not achieve favorable attenuation of iPTH levels, probably because secondary hyperparathyroidism aggravated over time.

Our study has several limitations. Firstly, this study was a single-center study with limited sample size, although the number of screened patients was relatively large. Secondly, because the compliance with efficient treatment of hyperphosphataemia included in the treatment group was voluntary and wealthier patients who were willing to pay for the phosphate binders required for the treatment, whereas the control group will presumably contain less wealthy patients, the study was not a double-blinded, randomized placebo-controlled trial. Thirdly, the factor in a will also probably mean that the diets of the treatment versus control groups would be different and this might explain why the baseline serum phosphorus concentration was different between the two groups. Finally, the prognosis of the patients, such as survival or mortality, was not analyzed in the study.

In conclusion, this study indicates that intensive treatment of hyperphosphatemia in patients on maintenance hemodialysis helps to decrease iPTH levels, especially for those who achieved a substantial reduction in serum phosphorus in a relatively short time period. The involvement of hyperphosphatemia in the pathogenesis of secondary hyperparathyroidism remains unclear and may require further investigation. Our results add to our knowledge that correction and prevention of hyperphosphatemia is of great importance for maintenance hemodialysis patients, especially at the early stage of hyperphosphatemia. In addition, we suggest that phosphorus binders should be covered under Chinese medical insurance, since they contribute to better management of hyperphosphatemia and improved quality of life for hemodialysis patients.
